# Fortification of Fermented Camel Milk with *Salvia officinalis* L. or *Mentha piperita* Leaves Powder and Its Biological Effects on Diabetic Rats

**DOI:** 10.3390/molecules28155749

**Published:** 2023-07-29

**Authors:** Magdy Ramadan Shahein, Mahmoud Ibrahim El-Sayed, Enrique Raya-Álvarez, Abdelmoneim Ahmed Elmeligy, Mohamed A. Mohamady Hussein, Murad A. Mubaraki, Ahmad Agil, Ehab Kotb Elmahallawy

**Affiliations:** 1 Department of Food Science and Technology, Faculty of Agriculture, Tanta University, Tanta 31527, Egypt; magdrsh10@gmail.com; 2 Department of Dairy Technology Research, Food Technology Research Institute, Agricultural Research Center, Giza 12622, Egypt; mahmoud.elsayed@arc.sci.eg; 3 Rheumatology Department, Hospital Universitario San Cecilio, Av. de la Investigación, s/n, 18016 Granada, Spain; enriraya@ugr.es; 4 Department of Pathology, Faculty of Veterinary Medicine, Suez Canal University, Ismailia 41522, Egypt; elmeligy52@yahoo.com; 5 Department of Pharmacology, Medical Research and Clinical Studies Institute, National Research Centre, 33 El Bohouth St., Dokki, Giza 12622, Egypt; almohammadeymr2023@gmail.com; 6 Clinical Laboratory Sciences Department, College of Applied Medical Sciences, King Saud University, Riyadh 12372, Saudi Arabia; mmubaraki@ksu.edu.sa; 7 Department of Pharmacology, Biohealth Institute Granada (IBs Granada) and Neuroscience Institute, School of Medicine, University of Granada, 18016 Granada, Spain; aagil@ugr.es; 8 Departamento de Sanidad Animal, Grupo de Investigación en Sanidad Animal y Zoonosis (GISAZ), Facultad de Veterinaria, Universidad de Córdoba, 14004 Córdoba, Spain; 9 Department of Zoonoses, Faculty of Veterinary Medicine, Sohag University, Sohag 82524, Egypt

**Keywords:** camel milk, fermented milk, anti-diabetic, sage, mint, antioxidants, tandem mass spectrometry

## Abstract

The incorporation of fermented camel milk with natural additives possesses numerous benefits for the treatment of various pathological and metabolic conditions. The present study investigated the impact of fortification of fermented camel milk with sage or mint leaves powder (1 and 1.5%, respectively) on glucose and insulin levels, lipid profile, and liver and kidney functions in alloxan-induced diabetic rats. The gross chemical composition of sage and peppermint leaves powder was studied. The chemical composition of sage and mint extracts was performed using liquid chromatography–electrospray ionization–tandem mass spectrometry (LC-MS) of sage and mint extracts. Furthermore, a total of forty-two adult normal male albino rats were included in this study, whereas one group was kept as the healthy control group (*n* = 6 rats) and diabetes was induced in the remaining animals (*n* = 36 rats) using alloxan injection (150 mg/kg of body weight). Among diabetic rats groups, a control group (*n* = 6 rats) was kept as the diabetic control group whereas the other 5 groups (6 rats per group) of diabetic rats were fed fermented camel milk (FCM) or fermented camel milk fortified with 1 and 1.5% of sage or mint leaves powder. Interestingly, the oral administration of fermented camel milk fortified with sage or mint leaves powder, at both concentrations, caused a significant decrease in blood glucose level and lipid profile, and an increase in insulin level compared to the diabetic control and FCM groups. Among others, the best results were observed in the group of animals that received fermented camel milk fortified with 1.5% sage powder. In addition, the results revealed that the fermented camel milk fortified with sage or mint leaves powder improved the liver and kidney functions of diabetic rats. Our study concluded that the use of sage and mint leaves powder (at a ratio of 1.5%) with fermented camel milk produces functional food products with anti-diabetic activity.

## 1. Introduction

Diabetes mellitus (DM) is a chronic metabolic condition described by persistent hyperglycemia due to the incapability of the pancreas to produce enough insulin, or the organ cannot use the produced insulin (insulin resistance), or a combination of both [1,2]. The available data from IDF confirmed that, in 2021, the number of people (20 to 79-year-olds) suffering from diabetes was predestined to be near 537 million [3]. This number is foreseeable to reach 643 million in 2030 and 783 million by 2045. Moreover, diabetes increases the serum level of urea and creatinine [4]. According to the most recent statistics from the Food and Agriculture Organization (FAO), the camel world population is estimated to be around 32.6 million [5]. Camel’s milk is a vital part of the staple diet in several parts of the world, especially in the arid and semi-arid zones. Camel’s milk is rich in health-beneficial substances, such as lactoferrin, lysozyme, lactoperoxidase, bioactive peptides, mono and polyunsaturated fatty acids, minerals (calcium, magnesium, copper, iron, zinc, phosphorous, potassium, and sodium), immunoglobulins and vitamins including, B1, B2 and C [6,7,8,9]. Camel milk has been known as a source for the production of dairy products with excellent therapeutic properties such as fermented milk [10]. Raw and fermented camel milk is found to have many health benefits such as anticancer, antimicrobial, antioxidant, anti-inflammatory, anti-diabetic, anti-diarrhea, hypocholesterolemic, and angiotensin I-converting enzyme (ACE) inhibitory activities [11,12,13,14]. It was reported that camel milk is a unique source of nutrients and is considered a superfood with high medicinal values [1,9,15,16]. Camel milk has been shown to improve other pathophysiological aspects related to diabetes as a chronic disease such as obesity, insulin resistance, wound healing and inflammation [17,18]. Camel milk improves diabetes complications such as wounds, kidney and liver failures, and oxidative stress. Also, camel milk improves diabetes complications such as liver and kidney failures, wounds, and oxidative stress [19]. In a study performed by Meena et al. (2016) [17], camel milk intake could enhance the level of antioxidative enzymes (catalase, glutathione peroxidase, and superoxide dismutase) in the pancreas in diabetic rats and, thus, could ameliorate hyperglycemia and oxidative damage. Furthermore, a previous study [20] found that raw camel milk caused an increase in insulin secretion, and reduced about 30–35% of required insulin in type 1 diabetes patients.

Fortifications of dairy products with natural substances enhance the physicochemical characterizations and nutritive values of functional food, and therefore, have attracted many research projects over the last few decades [21,22,23,24,25,26,27,28,29]. Many studies reported that herbal therapy is considered an effective way to the management of diabetes. These plants contain phenolics, flavonoids, carotenoids, alkaloids, terpenoids, and glycosides and may exert anti-diabetic effects to some extent [30,31]. Despite its traditional applications in food flavoring, *Mentha* spp. are widely used for treating not only fever and cold but also cardiovascular and gastrointestinal disorders as folk medicines [32]. Rajeshwari et al. 2012 [33], reported that the administration of mint leaves powder (5 g/day) to type 2 diabetes patients for 60 days reduced oxidative stress by decreasing lipid peroxidation, protein oxidation, increased serum beta carotene, and vitamin A, E, and C levels. Among others, the anti-diabetic actions of natural plant products are expressed by their role in activation of the antioxidant enzymes such as catalase, glutathione peroxidase, and superoxide dismutase [34,35], where the level of antioxidant enzymes such as superoxide dismutase was elevated in the presence of mentha extract [36]. In addition, it improved the activity of some antioxidant enzymes, i.e., glutathione-S-transferase (GST), in addition to the content of reduced glutathione (GSH). Chandirasegaran et al., 2014 [37] detected a significant decrease in blood glucose and creatinine levels as well as an increase in insulin levels of diabetic rats after being treated with mint (300 mg/kg B.W) for 45 days. These findings determined that mint possesses anti-diabetic activity against streptozotocin-induced diabetic rats. Sage, *Salvia officinalis* L., is well-reputed to cure diabetes or restrain its complications [38]. A previous study [39] found that the treatment of alloxan-induced diabetic rats with aqueous and ethanol extracts of *Salvia officinalis* leaves at a concentration of 100 mg/kg B.W for 14 days decreased the levels of blood glucose, triglycerides, and total cholesterol. The suggested mechanisms for anti-diabetic actions of salvia species extracts are the increase in insulin sensitivity, activation of pancreatic b-cells, and peripheral use of glucose, and the inactivation of insulinase enzyme and the glycogenolysis reduction decreases the absorption of glucose from the intestine and increases the synthesis of glucose in the liver [40]. The protective effect of sage extract against the depletion of antioxidant enzymes may contribute to its anti-diabetic effects [39]. Limited information is available about the biological effects of fermented camel milk fortified with sage and mint (*Mentha piperita*) leaves powder on alloxan-induced diabetic rats. The present study aimed to evaluate the effects of camel milk fortified with sage and mint leaves powder on the biochemical markers of alloxan-induced diabetic rats.

## 2. Results

### 2.1. Proximate Chemical Composition of Peppermint and Sage Leaves Powder and Their Antioxidant Activity

The proximate chemical composition of peppermint and sage leaves powder is shown in Table 1. Many studies reported that the plant extracts were used effectively to treat diabetic animals, where their components could induce one or more of the following actions: antioxidant, antihyperglycemic, increasing release of insulin, and anti-glycation agents [41,42,43,44]. Data in Table 2 showed the antioxidant activity of mint and sage extracts. The results found that sage extract was higher in total phenolic contentment (7.35 mg GAE/g) than mint extract (6.60 mg GAE/g), while the mint extract was the highest in total flavonoids (184 µg/mL). Moreover, a higher DPPH scavenging activity (%) was found with sage extract, while a higher FRAP value was observed with mint extract.

### 2.2. Chemical Composition of Sage and Mint Extracts Using Liquid Chromatography–Electrospray Ionization–Tandem Mass Spectrometry of Sage and Mint Extracts (LC-MS)

As shown in Table 3, the potential bioactive compounds of both SE and ME were identified using LC-ESI-MS/MS. As depicted in Table 3, twenty compounds were identified by comparing the fragmentation peaks with those of the corresponding standards. Out of them, 16 identified compounds of SE and ME were found and comprise chlorogenic acid, gallic acid, caffeic acid, rutin coumaric acid, naringenin, quercetin, ellagic, 3.4-dihydroxybenzoic acid, acid hesperetin, methyl gallate, kaempferol, ferulic acid, syringic acid, apigenin, and luteolin.

### 2.3. Effect of Fermented Camel Milk on Alloxan-Induced Diabetic Rats

#### 2.3.1. Serum Glucose and Insulin Determination

**Figure 1:**
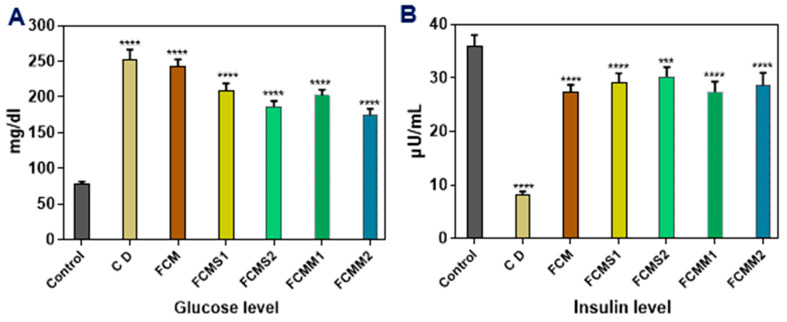
Effect of fermented camel milk fortified with sage and mint leaves powders on (**A**) glucose level and (**B**) insulin level of normal and diabetic rats. Control: normal healthy rats; CD: control diabetic rats; FCM: fermented camel milk; FCMS1: fermented camel milk with 1.0% sage leaves powder; FCMS2: fermented camel milk with 1.5% sage leaves powder; RCMM1: fermented camel milk with 1.0% mint leaves powder; FCMM2: fermented camel milk with 1.5% mint leaves powder. Data are expressed as average ± SD (*n* = 6). *** *p* < 0.0002, **** *p* < 0.0001, versus control.

The anti-diabetic properties of camel milk are very complex, involving many cellular and molecular mechanisms and aspects of metabolism and transport of glucose as well as the synthesis and secretion of insulin [45,46]. Data presented in Figure 1 showed the effect of fermented camel milk fortified with sage and mint leaves powders by ratio 1 and 1.5% on plasma glucose and insulin levels of diabetic rats. Results indicated that a higher plasma glucose level (253 mg/dL) was observed in the diabetic control group. On the other hand, the oral intake of fermented camel milk with or without fortification by sage and mint leaves powders significantly (*p* < 0.05) decreased the plasma glucose level in diabetic rats, while the normal rats were not affected. The oral intake of fermented camel milk fortified with saga and mint powder (FCMM1, FCMM2, FCMS1, and FCMS2) caused a significant decrease in the plasma glucose level compared with the group of fermented camel milk (FCM), and a higher decrease was found with FCMM2 and FCMS2 groups (186.3 and 175.2 mg/dL, respectively). The induction with alloxan caused a significant (*p* < 0.05) decrease in the insulin level in rats’ plasma (Figure 1). On the other hand, the oral intake of fermented camel milk with or without sage and mint powder significantly (*p* < 0.05) increases the insulin level in the blood again. The results showed that higher insulin levels were observed in the healthy control group (35.9 µU/mL), followed by the animal groups’ intake of fermented camel milk fortified with 1 and 1.5% sage powder (29.11 and 30.2 µU/mL, respectively), while no significant (*p* > 0.05) differences were found between FCM, FCMM1, and FCMM2 groups (27.3, 27.4, and 28.6 µU/mL, respectively).

#### 2.3.2. Lipid Profile

**Figure 2:**
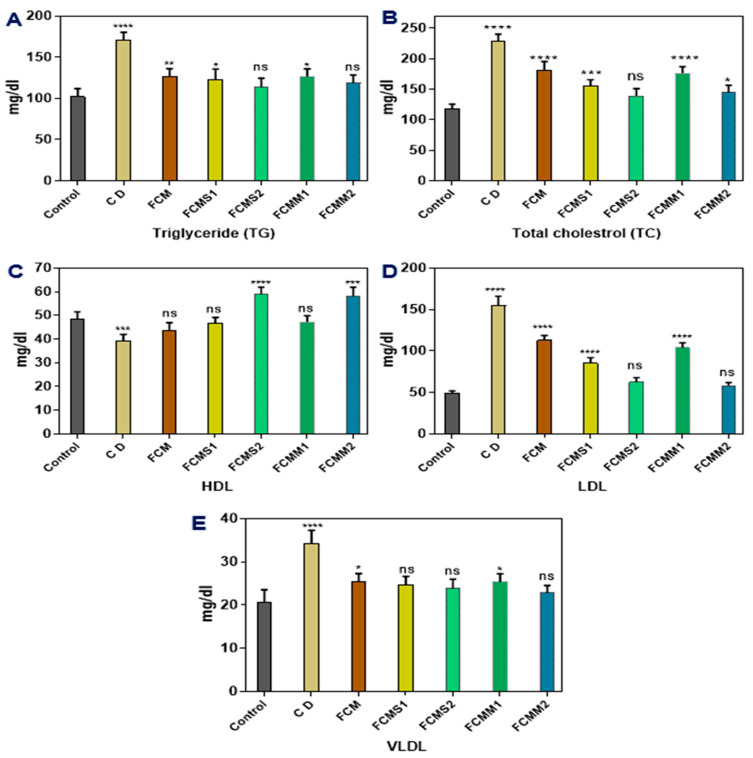
Effect of fermented camel milk fortified with sage and mint leaves powders on lipid profile in plasma of normal and diabetic rats (**A**, **B**, **C**, **D**, and **E**, respectively). Control: normal healthy rats; CD: control diabetic rats; FCM: fermented camel milk; FCMS1: fermented camel milk with 1.0% sage leaves powder; FCMS2: fermented camel milk with 1.5% sage leaves powder; RCMM1: fermented camel milk with 1.0% mint leaves powder; FCMM2: fermented camel milk with 1.5% mint leaves powder. Data are expressed as average ± SD (*n* = 6). * *p* < 0.0332; ** *p* < 0.0021, *** *p* < 0.0002, **** *p* < 0.0001, versus control.

Data in Figure 2 showed that after eight weeks of animal induction using alloxan, the animal untreated with fermented camel milk (diabetic control) displayed an increase in plasma total triglyceride (TG) and total cholesterol (TC), compared with healthy control and animal groups treated with fermented camel milk (FCM with or without mint and sage powders, FCMS and FCMM). The results showed that the oral administration of FCM or FCMS and FCMM significantly decreased TG and TC in diabetic rats groups. A higher decrease in TG and TC levels was found in FCMS2 and FCMM2 groups compared to FCMS1, FCMM1, and FCM groups. The results determined that the oral administration of fermented camel milk (FCM) or fermented camel milk fortified with 1 and 1.5% of sage or mint powder (FCMS and FCMM) caused a significant decrease in low-density lipoprotein cholesterol (LDL-c) and very low-density lipoprotein cholesterol (VLDL-c) levels compared with diabetic control group, while the high-density lipoprotein cholesterol (HDL-c) was significantly increased. The higher decrease in LDL values was found with FCMS2 and FCMM2 groups and there were no significant differences between the two groups, while the lowest value of VLDL was observed with FCMM2. Also, higher values of HDL were found in FCMM2 and FCMS2 groups compared with all other groups. It seems that the oral administration of fermented camel milk fortified with sage and mint powder by a ratio of 1 and 1.5% improved the lipid profile of diabetic rats, and the best results were found with an additional ratio of 1.5% of each herpes powder.

### 2.4. Liver Functions

**Figure 3:**
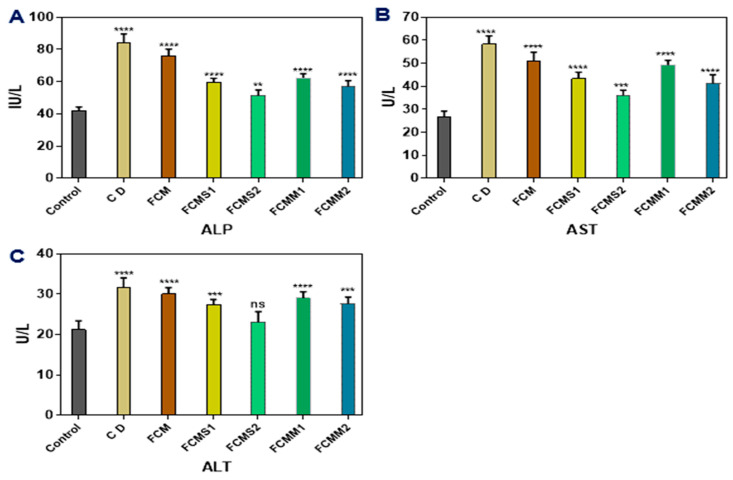
Effect of fermented camel milk fortified with sage and mint leaves powders on liver enzymes in plasma of normal and diabetic rats (**A**, **B,** and **C**, respectively). Control: normal healthy rats; CD: control diabetic rats; FCM: fermented camel milk; FCMS1: fermented camel milk with 1.0% sage leaves powder; FCMS2: fermented camel milk with 1.5% sage leaves powder; RCMM1: fermented camel milk with 1.0% mint leaves powder; FCMM2: fermented camel milk with 1.5% mint leaves powder. Graphs show mean values ± SD (*n* = 6). Data are expressed as average ± SD (*n* = 6). ** *p* < 0.0021, *** *p* < 0.0002, **** *p* < 0.0001, versus control.

In the current study, treatment of diabetic rats with fermented camel milk’s for 8 weeks significantly improved liver functions as evidenced by the following observations. Induction of rats with alloxan alone (diabetic control group) caused a significant (*p* < 0.05) increase in ALP, AST, and ALT compared with the healthy control group (Figure 3). These increases in ALP and AST were significantly (*p* < 0.05) decreased after being treated with FCM (FCM group) and FCM fortified with 1 or 1.5% of sage and mint powder. Meanwhile, the values of ALT were significantly (*p* < 0.05) decreased in FCMS1, FCMS2, and FCMM2 groups, while FCM and FCMM1 were not affected, compared with the diabetic control group. The treatment with fermented camel fortified with 2% sage powder (FCMS2 group) reduced the increase in liver functions to be close to the normal range. No significant (*p* > 0.05) differences were observed between FCMS1 and FCMM2.

### 2.5. Kidney Functions

**Figure 4:**
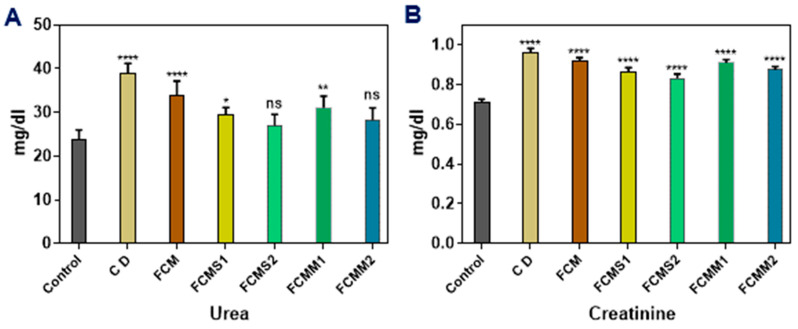
Effect of fermented camel milk fortified with sage and mint leaves powders on urea and creatinine levels of normal and diabetic rats (**A** and **B**, respectively). Control: normal healthy rats; CD: control diabetic rats; FCM: fermented camel milk; FCMS1: fermented camel milk with 1.0% sage leaves powder; FCMS2: fermented camel milk with 1.5% sage leaves powder; RCMM1: fermented camel milk with 1.0% mint leaves powder; FCMM2: fermented camel milk with 1.5% mint leaves powder. Data are expressed as average ± SD (*n* = 6). * *p* < 0.0332; ** *p* < 0.0021, **** *p* < 0.0001, versus control.

A serious complication of diabetes disease is diabetic nephropathy (DN), which is the most popular cause of chronic kidney disease, especially in Western countries, affecting 30–40% of patients with type 1 and type 2 diabetes [47]. As displayed in Figure 4, the induction of rats with alloxan significantly (*p* < 0.05) increased serum urea and creatinine levels and the high values were found in the positive control group as compared to the negative control group. Treatment with FCM or FCM fortified with sage and mint powder significantly decreased serum urea and creatinine levels. The higher decrease in urea level was observed with fermented camel milk samples containing 1.5% of sage or mint powder (FCS2 and FCMM2), followed by the samples containing 1% of sage and mint powder (FCS1 and FCMM1), and then samples of FCM alone. Concerning creatinine levels in diabetic rats groups, the results showed that the creatinine levels were significantly decreased administration of FCM, FCMS, and FCMM, and a higher decrease was found with FCMS1, FCMS2, and FCMM2, followed by FCMM1 and FCM groups.

## 3. Discussion

In the present study, we confirmed that the supplementation of fermented camel milk with sage and mint powder increased its anti-diabetic effects on alloxan-induced diabetic rats, whereas the oral administration of fermented camel milk fortified with sage and mint powder caused a significant decrease in blood glucose level and lipid profile and increase in insulin level compared to the control (+) and FCM groups. In relation to the chemical composition of sage and mint powder, which is shown in Table 2, the results of LC-MS revealed higher contents of phenolic and flavonoids in the study. Quantitative LC-MS/MS analysis revealed that the most abundant compounds in ME in order from high to low were naringenin, caffeic acid, luteolin, dihydroxybenzoic acid, hesperetin, chlorogenic acid, and apigenin. While the most abundant compounds in SE were naringenin, caffeic acid, luteolin, chlorogenic acid, apigenin, dihydroxybenzoic acid, quercetin, and coumaric acid. According to LC-MS analysis, phenolic and flavonoid compounds were identified and quantified (µg/g) in sage and mint extract. The polyphenols and flavonoid contents in the SE and ME were clearly superior according to LC-MS analysis. Importantly, the levels of naringenin, as a flavanone (RT 14.9.0 min), accounted for 2137 µg/g in SE and 4933 µg/g in ME. Caffeic acid (RT 8.04 min) accounted for 394.8 µg/g in SE and 339.9 µg/g in ME, whereas luteolin acid (RT 8.04 min) accounted for 372.4 µg/g in SE and 281.5 µg/g in ME. The obtained LC-MS results could be consistent with a previous study that declared that SE is rich in phenolic acids such as caffeic acid, and in flavonoids, in particular luteolin [48]. Also, previous studies [49,50] pointed out that ME is rich in naringenin, caffeic acid, and hydroxybenzoic acid, which is similar to the LC-MS of ME in this study. Other compounds were found at low levels but still in considered amounts such as apigenin, chlorogenic acid, hydroxybenzoic acid, quercetin, and coumaric acid in SE, and hesperetin, apigenin, rutin, and gallic acid in ME. The anti-diabetic activity of the dosage sage and mint powder could be attributed to these three compounds. Naringenin, a flavanon which is found in high amount in sage, was demonstrated for its anti-diabetic properties though inducing glucose uptake [51]. Additionally, Naringenin was reported for its tangible role to protect β cells from cytokine-induced cell death [52]. Caffeic acid is a phenolic acid, was found in a considered amount in SE according to LC-MS, and might play an important role in the anti-diabetic activity of the dosage form, due to its antioxidant activity and the capability to reduce the glucose level, enhance glucose-stimulated insulin secretion and glucose sensitivity [53,54]. Moreover, caffeic acid could be an effective agent in alleviating diabetes and associated complications [55]. Luteolin and apigenin as flavone class (flavonoids) showed anti-diabetic effects through activating expression levels of insulin receptor substrate-1, insulin receptor substrate-2, and phosphatidylinositol 3-kinase/protein kinase B pathway in IR-HepG2 cells [56]. High-glucose-simulated diabetes is associated with excessive production of reactive oxygen species (ROS) and advanced glycation end-products (AGEs). The increased levels of ROS increase oxidative stress with subsequent tissue damage (cellular inflammasomes) and are associated with insulin resistance [57]. Most of the identified compounds either in SE or ME such as caffeic acid, naringenin, chlorogenic acid, gallic acid, rutin, coumaric acid, quercetin, and ferulic acid are well known for their antioxidant activity, and therefore, such compounds can counteract the insulin resistance and can induce the glucose uptake. Moreover, these compounds such as chlorogenic acid were reported for their anti-diabetic effect through enhancing the insulin secretion and sensitivity.

It should be noted that experimental induction of diabetes by alloxan increased the level of plasma glucose significantly over the control level as reported elsewhere [58]. Also, in alloxan-induced diabetic rats, the levels of triglyceride, total cholesterol, LDL, VLDL, ALP, AST, and ALT were increased as declared in the results. The hyperglycemia induced by alloxan contributes to oxidative imbalance and increases oxidative stress. It is noteworthy to mention that alloxan induces diabetes in the diabetic model through the generation of reactive oxygen species (ROS) in excess levels, where ROS could be generated in this reaction involving alloxan, and then the alloxan is converted to a reduced product of dialuric acid combined with inhibition for the synthesis and secretion of insulin [59,60]. These findings cleared the relationship between the antioxidant compounds and the secretion of insulin in the body. In our study, the oral administration of FCM fortified with mint or sage leaves powder increased the secretion of insulin in diabetic rats and this may be due to the antioxidant components in mint and sage. Many studies reported the relationship between the antioxidant components in medicinal herbs such as sage and mint and potential anti-diabetic properties. Menthol and other volatile compounds in the leaves of M. piperita may be responsible for antioxidant and antioxidant activities [61]. Also, mint (*M. piperita*) leaf extract possesses a high amount of phenolic content, flavonoid content, and flavonols. Rosmarinic acid, caffeic acid, and its derivatives, and chlorogenic are the main phenolic compounds of the genus *Mentha* as well as the presence of some salvianolic acids [6,62]. In vitro assays have shown free radical (hydroxyls radicals, nitric oxide, hydrogen peroxide radicals, superoxide radicals, and DPPH radical) scavenging activities of extracts from different *Mentha* spp. [63,64,65]. The obtained results in this study agree with Agawane et al. [65], who found that the methanolic leaves extract of *Mentha arvensis* L. showed the ability to scavenge DPPH free radicals which was found to be 78% at a concentration of 1000 mg/mL. The effect of antioxidative components on the inhibition of DPPH radicals is considered to be due to their ability to donate hydrogen [66].

The blood glucose level in diabetic rats was significantly decreased after administration of FCM, FCMS, and FCMM; this finding is in agreement with that found by Hussain et al., 2021 [67], who observed that the mean blood glucose in diabetic mice decreased from 346 (mg/dL) to 140 (mg/dL) after being treated with camel milk (83 mL/kg body weight for 7 weeks), which is not significantly different from the diabetic mice receiving glibenclamide (anti-diabetic drug) in a dose of 600 µg/kg body weight (blood glucose of 125 mg/dL). Also, Shori and Baba, 2014 [68] reported that fermented plain camel milk had higher anti-diabetic activity than fermented plain cow milk. The oral intake of camel milk (at a dose of 250 mL/24 h/15 rats) reduced the blood glucose level from 462.3 ± 37.8 to 96.7 ± 11.1 mg/dL [69], while oral administration of camel milk for three weeks decreased the level of blood glucose of alloxan-induced diabetic rats from 10.88 ± 0.55 to 6.22 ± 0.5 mmol/L [70]. On the same side, Hamad et al., 2011 [71] noted that camel milk had higher anti-diabetic activity (49%) compared with buffalo and cow milk (11%) in diabetic Sprague Dawley rats. Previous studies [72,73] observed that camel milk had a significant hypoglycemic effect when administered to type 1 diabetic patients as an adjunct therapy for 3 months. Also, Agrawal et al. 2005 [74] reported that camel milk as an adjunct to insulin therapy appears to be safe and efficacious in improving long-term glycemic control and helps in reduction in the doses of insulin in patients with type 1 diabetes. One of the suggested mechanisms of the anti-diabetic effect of camel milk might be attributable to the inhibition of various metabolic enzymes such as dipeptidyl peptidase IV [DPP-IV, an enzyme that degrades the insulin-secreting incretin hormones gastric inhibitory polypeptide (GIP) and glucagon-like peptide (GLP), α-glucosidase and α-amylase [75]. The potential inhibition of DPP-IV is due to bioactive peptides resulting after hydrolysis of camel milk proteins throughout proteolysis or fermentation process [12], particularly bioactive peptides released from whey proteins [76,77]. Additionally, the presence of hydrophobic amino acids in the bioactive peptides is considered an additional factor for DPP-IV inhibition because these amino acids may further enhance interaction with the active site of DPP-IV [78,79]. Another study suggested that the anti-diabetic activity of camel milk due to its effect on the insulin receptors [80], while Mehaia et al., 1995 [81] reported that the content of insulin-like proteins in camel milk was three times more than in cow milk.

In the present study, it was observed that the corporation between sage or mint powder and fermented camel milk increased anti-diabetic activity (decreased glucose level and increased insulin level in blood plasma). This could be attributed to the additional biological activity of mint or sage extract as an antioxidant and anti-diabetic agent along with fermented camel milk. According to previous studies, the anti-diabetic activity of sage leaves powder due to its activity in reduced the blood glucose level and also inhibits the activity of the intestinal maltase and sucrase enzymes [39,82]. A previous study [83] found that the oral administration of peppermint juice for 21 days significantly (*p* < 0.0010) decreased the blood glucose level in alloxan-induced diabetic rats. Diabetes is associated with an increase in oxidative stress as shown by an increase in free radicals and decreased activities of catalase (CAT), superoxide dismutase (SOD), glutathione S-transferase (GST), glutathione peroxidase (GPX), and GSH [84]. Free radicals play an important role in the development of both type I and type II diabetes [85]. A previous work [86] reported that the elevation in plasma insulin levels in the sage extract-treated STZ diabetic rats could be due to substances present in the plant extract which stimulate insulin secretion or which protect the intact functional b-cells from further deterioration so that they keep active and continue to insulin production. The same study [86] showed that the methanol extract of *S. officinalis* causes a significant reduction in glucose concentrations on STZ -induced hyperglycemic rats. Also, a previous study [39] found that the alloxan-induced diabetic rats treated with aqueous and ethanol extracts (100 mg/kg) of sage (*Salvia officinalis*) leaves showed a significant reduction (*p* < 0.05) in fasting blood glucose. The effects of plants on diabetes disease were summarized as increasing insulin secretion, increasing glucose uptake by fat tissues and skeletal muscle, inhibiting the production of liver glucose, and inhibiting the absorption of glucose in the intestinal [40]. These results in agreement with previous work [69] noted that the oral administration of camel milk reduced the increase in TG, TC, LDL-C, and VLDL-C in diabetic rats compared with the diabetic control group. A previous study [87] evaluated the effects of camel milk on the TC, HDL, and TG levels in type 1D and type 2D, respectively, and their findings agreed with our results that camel milk normalized the alteration in TG and HDL-c, while reducing the increase in total cholesterol (TC) levels. Therefore, camel milk can give promising results when used as a dietary supplement for patients of type 1D. In the same line, Khattab et al., 2012 [88] found that treated diabetic rats with sage leaves induced significant improvement in lipid profile parameters as compared with the non-treated diabetic group and concluded that sage had a potent hypoglycemic activity and related this effect to its antioxidant activities. Regulation of the levels of cholesterol and triglyceride in the blood is an important way to protect humans from coronary heart disease. It was found that administration of sage infusion for 12 weeks reduced total cholesterol, triglycerides, and low-density lipoprotein (LDL-c) in rats, while HDL-c was increased [89]. Also, a previous study [39] indicated that ethanolic and water extracts of sage leaves significantly lowered cholesterol and TG levels. Moreover, many studies cleared the significant role of mint leaves on diabetic rats. This hypolipidemic effect of sage may be related to the inhibition of hepatic de novo synthesis or the activation of β-oxidation [89]. A previous study [90] reported that treatment of diabetic rats with *M. piperita* caused a reduction in the levels of cholesterol, LDL-c, and triglycerides and increase the levels of HDL-c. Also, a previous work [91] found that treatment of hyperlipidemic rats with aqueous extract of Mentha piperita leaves extract for 21 days significantly reduced serum total cholesterol, triglycerides, and LDL-c, and was associated with a significant increase in HDL-c levels and decrease in the atherogenic index in indicating its potent anti-hyperlipidemic and antiatherogenic activity.

It should be stressed that AST, ALT, and LDH are enzymes mainly found in hepatocyte cytosol and cell membrane. They are good markers considerably used to evaluate hepatotoxicity and integrity of the membrane [92]. The increase in activities of plasma ALT, AST, ACP, ALP, and LDH means that diabetes caused hepatic dysfunction. Therefore, the increment of the activities of ALT, AST, ACP, ALP, and LDH in plasma may be mainly due to the leakage of these enzymes from the liver cytosol into the bloodstream which gives an indication of the hepatotoxic effect of alloxan [93,94]. A previous report [92] noted that the increase in liver enzyme activities in diabetic rats was reduced after being treated with sage essential oil. Similarly, in alloxan diabetic rats, ALT, AST, and ALP activities were superior to those in normal rats, but recovered after oral administration of fermented camel milk fortified with sage and mint powder. These results are similar to that found in a previous study [86], which reported that the recovery of liver cell integrity was obtained after treatment by sage. Oral administration of ethanolic extract of sage leaves to diabetic rats, lowered serum glucose, triglycerides, total cholesterol, urea, creatinine, AST, ALT, and enhanced plasma insulin, depending on the increasing dose [42].

Induction of hyperglycemia caused an increase in serum creatinine and urea levels, excessive proteinuria, and marked deterioration of kidney function, and microscopic examination of sections of the kidneys of diabetic animals showed pathological features of glomerulosclerosis, with abnormal extracellular matrix (ECM) accumulation, glomerular matrix expansion, tubular alveolar degeneration, and fibrosis, and increased urinary excretion [1]. The observed increase in serum creatinine, urea, and uric acid of diabetic animals compared with the nondiabetic control group agrees with previous work [42], while the consumption of camel milk caused a significant decrease in creatinine and urea in diabetic rats and this could be attributed to the hypoglycemic and antioxidant effects of camel milk [1]. The reported powerful hypoglycemic action of camel milk in diabetic patients is hypothesized to abolish the glucose-driven metabolic pathways. Intensive glycemic control in type 1 and type 2 diabetes mellitus patients results in a decrease in microalbuminuria. So, the observed renal protective effects of camel milk treatment in diabetic rats could be assigned to the glucose homeostatic action of camel milk. Our results were in accord with the earlier findings [72], which found a significant reduction of microalbuminuria in type 1 diabetes mellitus patients receiving camel milk, along with their standard anti-diabetic therapy suggesting a direct protective effect of camel milk against diabetic nephropathy [67,95,96].

The increase in serum alanine aminotransferase (ALT) and aspartate aminotransferase (AST) activities may indicate liver tissue damage, probably by altered cell membrane permeability leading to the leak of the enzymes from the tissues to the serum. Alanine and aspartate aminotransaminases are considered to be sensitive indicators of hepatocellular damage and within limit can provide a quantitative evaluation of the degree of damage to the liver [97]. Diabetes has a strong relationship with renal and liver diseases [98]. Camel milk was reported to protect the liver and kidney function from failure. A previous study [1] found that the administration of camel milk to the control animals caused insignificant changes in the glomerulotubular morphology in comparison to the non-camel milk-treated control animals. Furthermore, the kidney slices obtained from the diabetic animals and stained with the hematoxylin and eosin showed glomerular expansion and tubular alveolar degeneration. A previous work [99] mentioned that the induction with streptozotocin caused damage to the kidney tissue of diabetic rats, and the untreated group showed severe glomerular necrosis with lymphocyte hyperplasia when compared with the normal. This result is similar to the work carried out by Trujillo et al. [100], who reported that abnormal levels of serum urea usually signify decreased renal function, so plasma urea is a recognized marker of glomerular filtration rate (GFR) and in nephropathy.

## 4. Material and Methods

### 4.1. Materials and Chemicals

Camel milk (total solids 11.84%, protein 3.22%, fat 3.43%, pH 6.60, and acidity 0.175%) was obtained from a private farm in El-Arish, North Sinai Governorate, Egypt. Commercially available lyophilized culture (Yo-fast 88, contains *Streptococcus thermophillus* and *Lactobacillus delbrueckii* ssp. *bulgaricus*) was purchased from Chr. Hansen Laboratories, Hoersholm, Denmark. Mint (*Mentha piperita*) and sage (*Salvia officinalis*) leaves were obtained from El-Arish local market, North Sinai Governorate, Egypt. Alloxan monohydrate, analytical reagent grade purchased from Sigma Chemical Co. (Sigma-Aldrich Company Ltd., Gillingham, UK). 1, 1-dipheny l-2-picryl-hydrazyl (DPPH) was purchased from Sigma-Aldrich (Munich, Germany). Potassium ferricyanide, ferric chloride, and gallic acid were purchased from Loba Chemie, Mumbai, India. Formic acid (LC grade) was provided by Fisher Chemical. Methanol and acetonitrile (LC grade) were supplied from Supelco, where Milli-Q water was used.

### 4.2. Methods

#### 4.2.1. Preparation of Mint and Sage Leaves Powder

The leaves of mint and sage were dried at 30–40 °C by the hybrid solar convective drying system (C.C.P. Parma—Parma, Italy), and then the leaves were ground until they become a powder.

#### 4.2.2. Preparation of Sage and Mint Extracts for Proximate Chemical Analysis

Five grams of mint and sage leaves powders were mixed with 100 mL ethanol solution 75%, stirring for 2 h at room temperature. Finally, the mixtures were filtered by Whatman No. 1 and the extracts were stored at 4 °C until analysis [101]. Furthermore, the gross chemical compositions of sage and peppermint leaves powder used for different treatments of fermented camel milk samples were determined and analyzed for moisture, protein, fat, and ash. Crude fiber contents were determined, while carbohydrate was calculated by difference according to the standard methods of the association of official analytical chemists [102].

#### 4.2.3. Preparation of Standard Compounds and Extract Samples Solutions and Identification of Their Chemical Composition of Sage and Mint Extracts and Their Compounds Using LC-ESI-MS/MS

Stock standard solutions in 1 mg/mL of standard compounds were prepared in methanol. Then, the stock solutions of each standard were diluted to 10 µg/mL and kept at 4 °C. The working standards mixture solution (200 ppb) was prepared by mixing 20 µL of each stock solution and then completing the volume to 1 mL using methanol. Then, 3 mg of the dried extract (mint extract or sage extract) was dissolved in 1 mL 80% methanol, sonicated for 15 min, filtered through a 0.45 µm syringe filter, and finally injected into LC-MS/MS. A blank 80% methanol was injected before each sample. The analysis of the extracts of sage and mint extracts was performed using liquid chromatography–electrospray ionization–tandem mass spectrometry (LC-ESI-MS/MS) with an ExionLC AC system for separation and SCIEX Triple Quad 5500+ MS/MS system equipped with electrospray ionization (ESI) for detection [103]. MRM transitions and the optimized mass spectrometer parameters of each compound are listed in Supplementary Table S1 and LC-ESI-MS spectra of standards and extracts are shown in Supplementary Figures S1–S3. The chromatographic conditions were optimized where the separation was performed using ZORBAX Eclipse Plus C18 Column (4.6 × 100 mm, 1.8 µm). The mobile phases consisted of two eluents, A: 0.1% formic acid in water; B: acetonitrile (LC grade)**.** The mobile phase elusion was programmed as follows: 2% B from 0–1 min, 2–60% B from 1–21 min, 60% B from 21–25 min, and 2% B from 25.01–28 min. The flow rate was optimized at 0.8 mL/min and the injection volume was 3 µL. Positive and negative ionization modes were applied in the same run for MRM analysis of the selected standard compounds, with the following parameters: curtain gas: 25 psi; ion spray voltage: 4500 and −4500 for positive and negative modes, respectively; source temperature: 400 °C; ion source gas 1 and 2 were 55 psi with a declustering potential: 50; collision energy: 25; collision energy spread: 10.

#### 4.2.4. Antioxidant Activity of Sage and Mint Extracts

##### Determination of Total Phenolic Contents (TPC) of Sage and Mint Extracts

The total phenolic contents of sage and mint extracts were determined according to the method of Abirami et al., 2014 [104]. Total phenol content (TPC) was expressed as Gallic acid equivalent (mg GAE/g plant material or extract).

##### Determination of Total Flavonoids (TF)

The TF content of sage and mint extracts was determined based on the method of Barros et al., 2011 [105]. A calibration curve of Rutin was prepared and TF content was determined.

##### DPPH Scavenging Activity %

The scavenging activity of 1,1-diphenyl-2-picrylhydrazyl (DPPH) radical was determined according to the method of Lim and Quah, 2007 [106]. Triplicate tubes were prepared for each extract. The results were expressed as % radical scavenging activity.

$$Radical\;scavenging\;activity\%=\frac{\left(A_{control}-A_{sample}\right)}{A_{control}}\times 100$$

IC_50_, which denotes the amount (mg) of the plant powder in 1 mL solution required to reduce the initial concentration of DPPH radicals by 50%, was also calculated. Ascorbic acid was used as a standard.

##### Ferric Reducing Antioxidant Power (FRAP)

The FRAP was determined according to the method of Oyaizu, 1986 [107]. Triplicate tubes were prepared for each extract. The FRAP values, expressed in mg GAE/g, were derived from a standard curve.

#### 4.2.5. Physicochemical Analysis of Camel Milk

Total solids (%), protein (%), and fat (%) of camel milk were determined using the AOAC procedures [108]. The pH of camel milk was measured using a digital pH meter (Martini, Italy). Titratable acidity (lactic acid %) of raw camel milk was evaluated by titration with NaOH (0.1 N) in the presence of phenolphthalein as an indicator. All analyses were performed in triplicate.

#### 4.2.6. Preparation of Fermented Camel Milk (FCMs)

Camel milk was divided into five portions. The first portion served as a control (FCM). Four portions of camel milk were supplemented with sage and mint leaves powder at levels of 1 and 1.5% (FCMS1 (1% sage), FCMS2 (1.5% sage), FCMM1 (1% mint), and FCMM2 (1.5% mint), and these percentages were chosen according to the sensory evaluation of fermented camel milk fortified with different percentages of these herbs (0.5, 1, 1.5, and 2%). Fermented milk was prepared according to Tamime and Robinson, 2007 [109]. Camel’s milk was heated at 72 °C/15 s, cooled to 40 °C, and then inoculated with 0.3% yogurt starter culture. Camel milk was incubated at 42 ± 1 °C until the pH value was decreased to approximately 4.6. The resultant fermented camel milk of all treatments was kept in a refrigerator (4 ± 1 °C) until use.

#### 4.2.7. Animals and Treatments

##### The Induction of Experimental Diabetes

Alloxan was dissolved in a saline solution (0.9% sodium chloride, pH 7). Diabetes was induced in normal healthy male albino rats by receiving an intra-peritoneal injection dose of alloxan 150 mg/kg body weight, according to the method described elsewhere [110]. After three days of the injection with alloxan, fasting blood samples were obtained to estimate fasting serum glucose higher than 200 mg/dL in rats, which were considered diabetes by the National Diabetes Data Group [111].

##### Experimental Design

Forty-two adult normal male albino rats of Sprague Dawley strain (140 ± 10 g) were obtained from the Vaccine and Immunity Organization, Ministry of Health, Helwan, Egypt. Animals were housed 6 per cage and fed on a basal diet prepared based on the American Institute of Nutrition [112] and consisting of 12% casein, 10% sugars, 10% sunflower oil, 1% vitamin mixtures, 4% mineral mixtures, 4% fiber, 58.50% starch, 0.3% DL-methionin, and 0.2% choline chloride, and given free access to fresh water ad libitum. Rats were acclimated for 2 weeks at 25 ± 1 °C with a 12 h dark and light cycle. The experimental period was 8 weeks after stabilization of diabetes for 1 week and the animals were divided into 7 major groups (6 rats per group) and treated based on the experimental protocol:

(1) healthy control (C group); (2) diabetic control (DC group); (3) diabetic + 85 mL FCM/kg B.W/day (FCM group); (4) diabetic + 85 mL FCMS1/kg B.W/day (FCMS1 group); (5) diabetic + 85 mL FMCS2/kg B.W/day (FCMS2 group); (6) diabetic + 85 mL FCMM1/kg B.W/day (FCMM1 group); and (7) diabetic + 85 mL FCMM2/kg B.W/day (FCMM2). The oral dose of fermented camel milk was chosen based on the study of Althnaian et al., 2013 [113]. At the end of the experimental period, rats fasted for 12 h, were anesthetized with ether, and killed. Fasting blood samples were collected in heparinized tubes from the killed animals and then centrifuged at 7200× *g* at 4 °C for 20 min (Sigma centrifuge 113, VWR International) to obtain plasma. The obtained plasma was stored at −80 °C until used for analyses [114].

##### Blood Biochemical and Enzymes Activities

Stored plasma samples were analyzed for plasma glucose concentration according to the method of Trinder [110] and National Diabetes Data Group [115]. Urea was determined as described elsewhere [116,117,118]. Creatinine was determined according to the method of Bartels and Böhmer, 1971 [119] and Fabiny and Ertingshausen, 1971 [120]. Triglycerides were determined according to the method of Bucolo and David, 1973 [121] and Fossati and Prencipe, 1982 [122]. Cholesterol was determined according to the method of Meiattini et al. 1978 [123]. High-density lipoprotein (HDL) cholesterol was determined according to the method of Grove, 1979 [124] and Burstein et al. 1970 [125]. Low-density lipoprotein (LDL) was determined by the calculation (cholesterol-(TG/5+HDL). Very low-density lipoprotein (VLDL) was calculated by dividing the values of TG by a factor of 5. The activities of plasma aspartate transaminase (AST) and alanine transaminase (ALT) were assayed by the method of Reitman and Frankel, 1957 [126]. Alkaline phosphatase (AlP) activity was determined in plasma according to the method of Belfield and Goldberg, 1971 [127]. Commercial kitts of the previous assays were obtained from Biosystems S.A. (Barcelona, Spain) (for glucose, cholesterol, HDL, TG, urea, and creatinine); QUIMICA CLINICA APLICADA S.A (Amposta, Spain) (for AST, ALT); and Biodiagnostic (ARE) (for ALP).

##### Determination of Blood Insulin Level

Insulin levels were estimated according to Abraham et al., 1978 [128] and Wilson and Miles, 1977 [129] using an ELISA kit by Linco Research Inc., Missouri, USA.

#### 4.2.8. Statistical Analysis

The results were analyzed using GraphPad Prism 8.0.2 software, GraphPad Software Inc, California, USA. The results were expressed as means ± SD, where the statistical significance was evaluated using one-way ANOVA. followed by Tukey’s correction (* *p* < 0.0332; ** *p* < 0.0021, *** *p* < 0.0002, **** *p* < 0.0001, versus control).

## 5. Conclusions

The current study concluded that supplementation of camel milk with sage and mint leaves powder ameliorated and normalized the changes in glucose, total cholesterol, and triglyceride levels in the blood of diabetic rats. The most marked findings were found with the fortification of fermented camel milk with sage leaves powder at a ratio of 1.5%. Given the above findings, it could be concluded that sage and mint leaves powder (at a ratio of 1.5%) can be used to produce healthy and functional fermented camel milk with high antioxidant activity and anti-diabetic activity. The presented promising data could be beneficial and encourage us to set up a further future study utilizing the major components in the fermented milk (e.g., bioactive peptides) with the major antioxidant components in sage or mint extracts to obtain the best dosage form with studying its effect on metabolites and key genes involved in the glucose metabolism pathway, as a potential promising anti-diabetic product in the future.

## Figures and Tables

**Table 1:** Proximate chemical composition of peppermint and sage leaves powder.

Composition%	Peppermint (*Mentha peperita*) Leaves Powder (%)	Sage (*Salvia officinalis*) Leaves Powder (%)
Ash	21	7.12
Crude	9.4	26.23
Lipids	5.68	7.91
Proteins	7.6	8.73
Carbohydrates	56.32	50.01

**Table 2:** Antioxidant activity of saga and mint leaves extracts.

Property	Sage	Mint
DPPH (%)	71.64 ± 3.45 ^a^	45.32 ± 3.45 ^b^
FRAP (mg GAE/g)	0.236 ± 0.008 ^b^	0.466 ± 0.041 ^a^
Total phenolic (mg GA/g)	7.35 ± 0.026 ^a^	6.60 ± 0.137 ^b^
Total flavonoids (µg/mL)	170.87 ± 4.04 ^b^	184.92 ± 4.96 ^a^

Mean values (±standard deviation), with different small letters, a and b, are significantly different at *p* < 0.05.

**Table 3:** LC-MS of mint extract and sage extract.

N	RT	Compound	Mint Extract µg/g	Sage Extract µg/g
1	7.35	Chlorogenic acid	40.07	155.47
2	12.9	Daidzein	ND	ND
3	3.89	Gallic acid	24.34	6.81
4	8.04	Caffeic acid	339.87	394.85
5	9.73	Rutin	25.47	0.15
6	9.53	Coumaric acid	15.43	36.12
7	9.5	Vanillin	ND	18.50
8	15.03	Naringenin	4933.35	2137.01
9	13.56	Quercetin	4.16	45.62
10	9.94	Ellagic acid	9.61	17.80
11	5.73	3.4-Dihydroxybenzoic acid	123.53	54.87
12	15.57	Hesperetin	81.01	2.61
13	14.1	Cinnamic acid	ND	ND
14	7.42	Methyl gallate	0.10	0.08
15	15.33	Kaempferol	0.29	9.99
16	10.21	Ferulic acid	10.91	17.26
17	8.37	Syringic acid	20.13	33.89
18	15.03	Apigenin	38.16	107.64
19	7.35	Catechin	ND	ND
20	13.5	Luteolin	281.54	372.40

ND: not detected.

## Data Availability

Data is contained within the article.

## Supplementary Materials

The following supporting information can be downloaded from: https://www.mdpi.com/article/10.3390/molecules28155749/s1. Table S1: MRM transitions and the optimized mass spectrometer parameters; Figures S1–S3: the mass liquid chromatography–electrospray ionization–tandem mass spectra of standards; sage and mint extracts are provided in the Supplementary file.

## List of Abbreviations

CM: camel milk; FCM: fermented camel milk; DM: diabetes mellitus; IDF: International Diabetes Federation; DPPH: 1, 1-diphenyl-2-picryl-hydrazyl; TPC: total phenol content; FRAP: ferric reducing antioxidant power; HDL-c: high-density lipoprotein cholesterol; LDL-c: low-density lipoprotein cholesterol; VLDL-c: very low-density lipoprotein cholesterol; TG: triglycerides; TC: total cholesterol; AST: aspartate transaminase; ALT: alanine transaminase; AlP: alkaline phosphatase.
