# Supplementary material for: Fortification of Fermented Camel Milk with Salvia officinalis L. or Mentha piperita Leaves Powder and Its Biological Effects on Diabetic Rats
Source: Molecules. 2023 Jul 29;28(15):5749. doi: 10.3390/molecules28155749 (PMC10420823; doi:10.3390/molecules28155749)
Supplement: Supplementary file 1 [file molecules-28-05749-s001.zip › molecules-2478720-supplementary.pdf]

molecules

Fortification of fermented camel milk with *Salvia officinalis L.* or *Mentha piperita* leaves powder and its biological effects on diabetic rats

Magdy Ramadan Shahein [1], Mahmoud Ibrahim El-Sayed [2], Enrique Raya-Álvarez [3], Abdelmoneim Ahmed Elmeligy [4], Mohamed A. Mohamady Hussein [5], Murad A. Mubaraki [6], Ahmad Agil [7], and Ehab Kotb Elmahallawy [8,9*]

[1] Department of Food Science and Technology, Faculty of Agriculture, Tanta University, 31527 Tanta, Egypt. magdrsh10@gmail.com (M.R.S)
[2] Department of Dairy Technology Research, Food Technology Research Institute, Agricultural Research Center, Giza, Egypt; mahmoud.elsayed@arc.sci.eg (M.I.E-S)
[3] Rheumatoloy Department, Hospital Universitario San Cecilio, Av. de la Investigación, s/n, 18016 Granada, Spain.; enriraya@ugr.es (E.R-A)
[4] Department of Pathology, Faculty of Veterinary Medicine, Suez Canal University, Ismailia, Egypt. elmeligy52@yahoo.com (A.A.E)
[5] Department of Pharmacology, Medical Research and Clinical Studies Institute, National Research Centre, 33 El Bohouth St., Dokki, Giza 12622, Egypt; almohammadeymr2023@gmail.com (M.A.M.H)
[6] Clinical Laboratory Sciences Department, College of Applied Medical Sciences, King Saud University, Saudi Arabia; mmubaraki@ksu.edu.sa (M.A.M)
[7] Department of Pharmacology, Biohealth Institute Granada (IBs Granada) and Neuroscience Institute, School of Medicine, University of Granada, 18016 Granada, Spain; aagil@ugr.es (A.A)
[8] Departamento de Sanidad Animal, Grupo de Investigación en Sanidad Animal y Zoonosis (GISAZ), Facultad de Veterinaria, Universidad de Córdoba, Córdoba, Spain; eehaa@unileon.es (E.K.E)
[9] Department of Zoonoses, Faculty of Veterinary Medicine, Sohag University, Sohag 82524, Egypt; eehaa@unileon.es (E.K.E)
Correspondence: eehaa@unileon.es (E.K.E.)

molecules

MDPI

Table S1. MRM transitions and the optimized mass spectrometer parameters.

Name	Q1 (m/z)	Q3 (m/z)	Expected RT (min)	CE (V)	CXP (V)	DP (V)
Gallic acid	168.9	124.9	3.9	-30	-11	-110
	168.9	79	3.9	-30	-11	-110
3.4-Dihydroxybenzoic acid	152.9	109	5.8	-40	-5	-75
	152.9	90.9	5.8	-20	-7	-75
Catechin	288.8	244.9	7.3	-16	-8	-40
	288.8	109	7.3	-32	-8	-40
Methyl gallate	183	124	7.5	-30	-10	-110
	183	140	7.5	-30	-10	-110
Chlorogenic acid	355.1	163	7.8	21	10	46
	355.1	89	7.8	75	14	46
Caffeic acid	178.9	135	8	-22	-9	-115
	178.9	107	8	-30	-7	-115
Syringic acid	196.9	122.8	8.4	-24	-5	-30
	196.9	181.9	8.4	-12	-5	-30
Coumaric acid	162.9	119	9.5	-20	-7	-90
	162.9	93	9.5	-40	-5	-90
Vanillin	151	136	9.6	-12	-9	-140
	151	92	9.6	-16	-7	-140
Rutin	609	299.9	9.7	-48	-15	-230
	609	270.9	9.7	-70	-9	-230
Ellagic acid	301	145	9.9	-40	-14	-120
	301	245	9.9	-38	-14	-120
Ferulic acid	192.8	133.9	10.2	-16	-5	-25
	192.8	177.9	10.2	-12	-5	-25
Daidzein	255.1	199	13.4	28	10	125
	255.1	91.1	13.4	44	10	125
Luteolin	284.7	132.9	13.5	-38	-10	-50
	284.7	150.9	13.5	-26	-10	-50
Querectin	301	151	13.6	-28	-9	-50
	301	178.8	13.6	-20	-7	-50
Cinnamic acid	146.9	102.6	14.2	-17	-6	-60
	146.9	77	14.2	-33	-6	-60
Naringenin	271	151	15	-24	-25	-130
	271	119	15	-34	-11	-130
Apigenin	269	151	15	-15	-7	-35
	269	117	15	-15	-7	-35
Kaempferol	284.7	93	15.3	-46	-10	-120
	284.7	116.8	15.3	-52	-10	-120
Hesperetin	301	164	15.6	-23	-10	-125

molecules

	301	136	15.6	-38	-10	-125

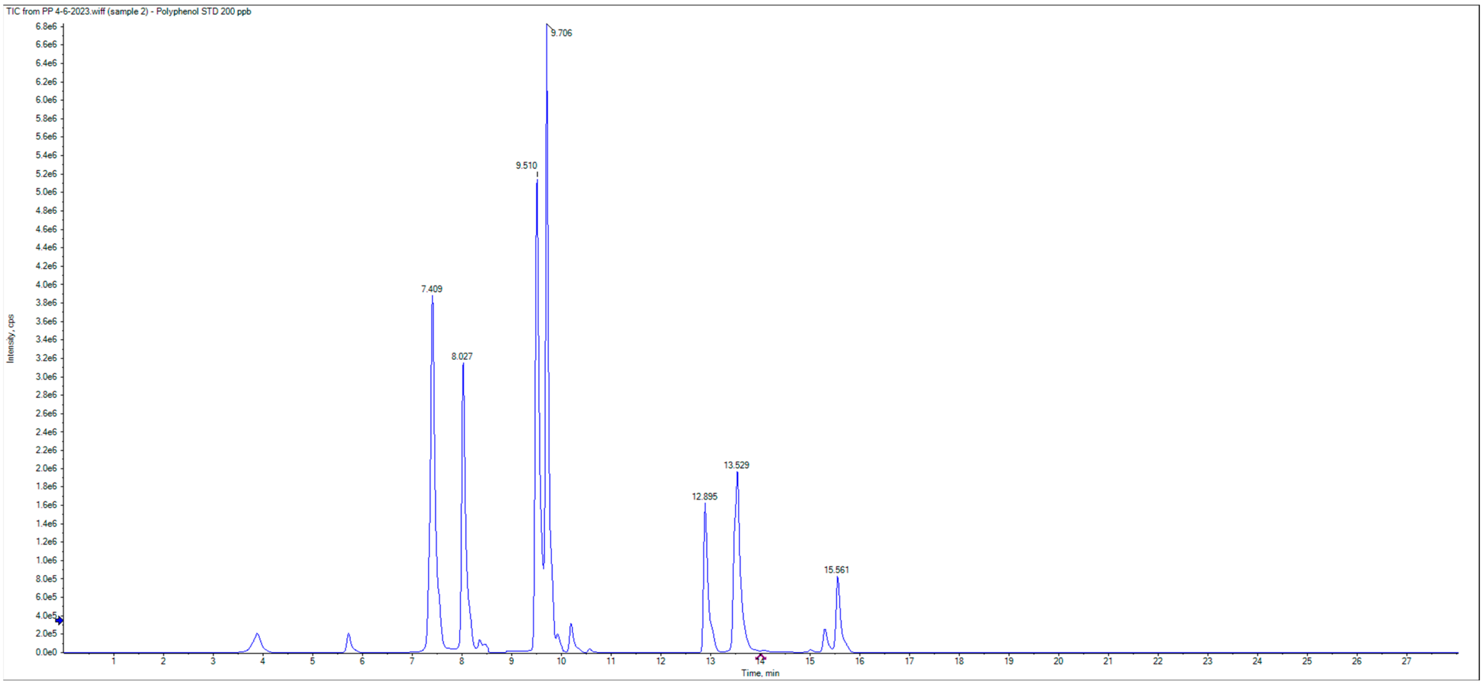

Figure S1. LC-ESI-MS spectra of standards.

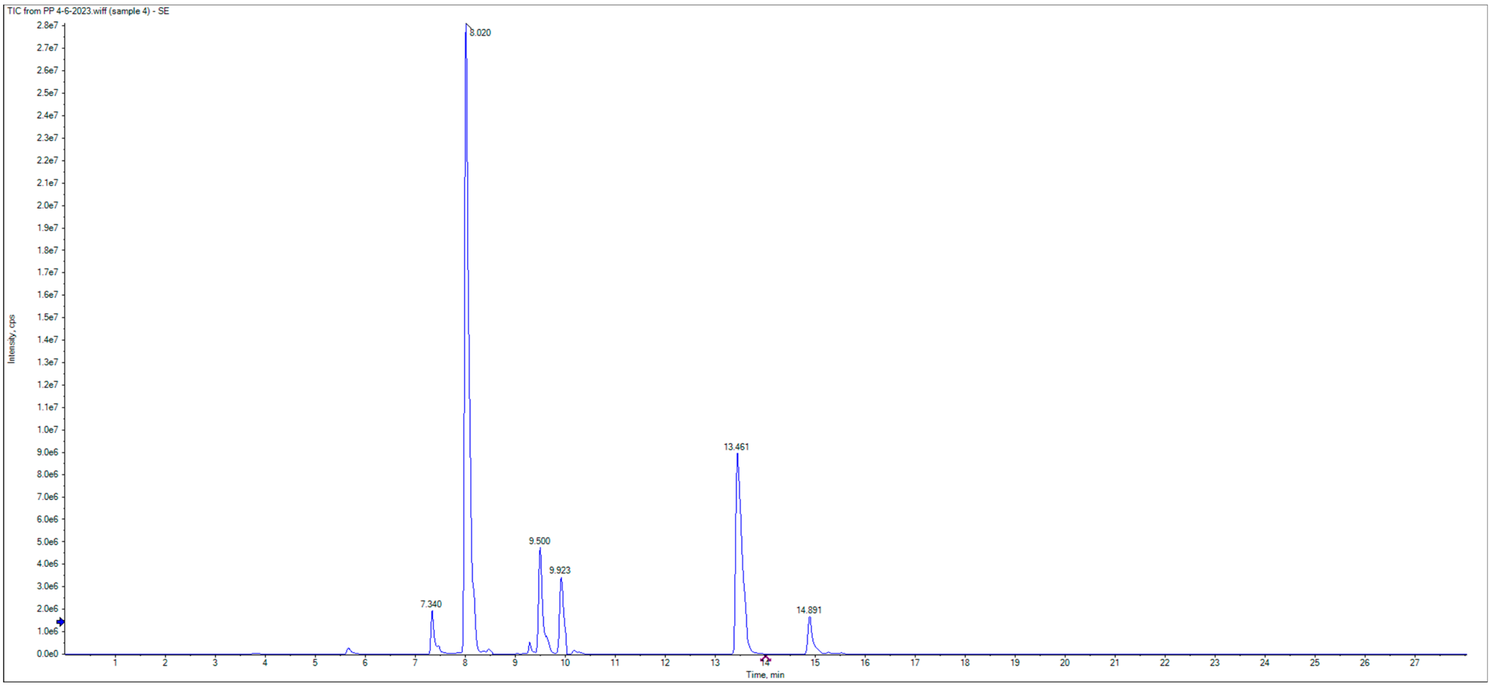

Figure S2. LC-ESI-MS spectra of sage extract.

molecules

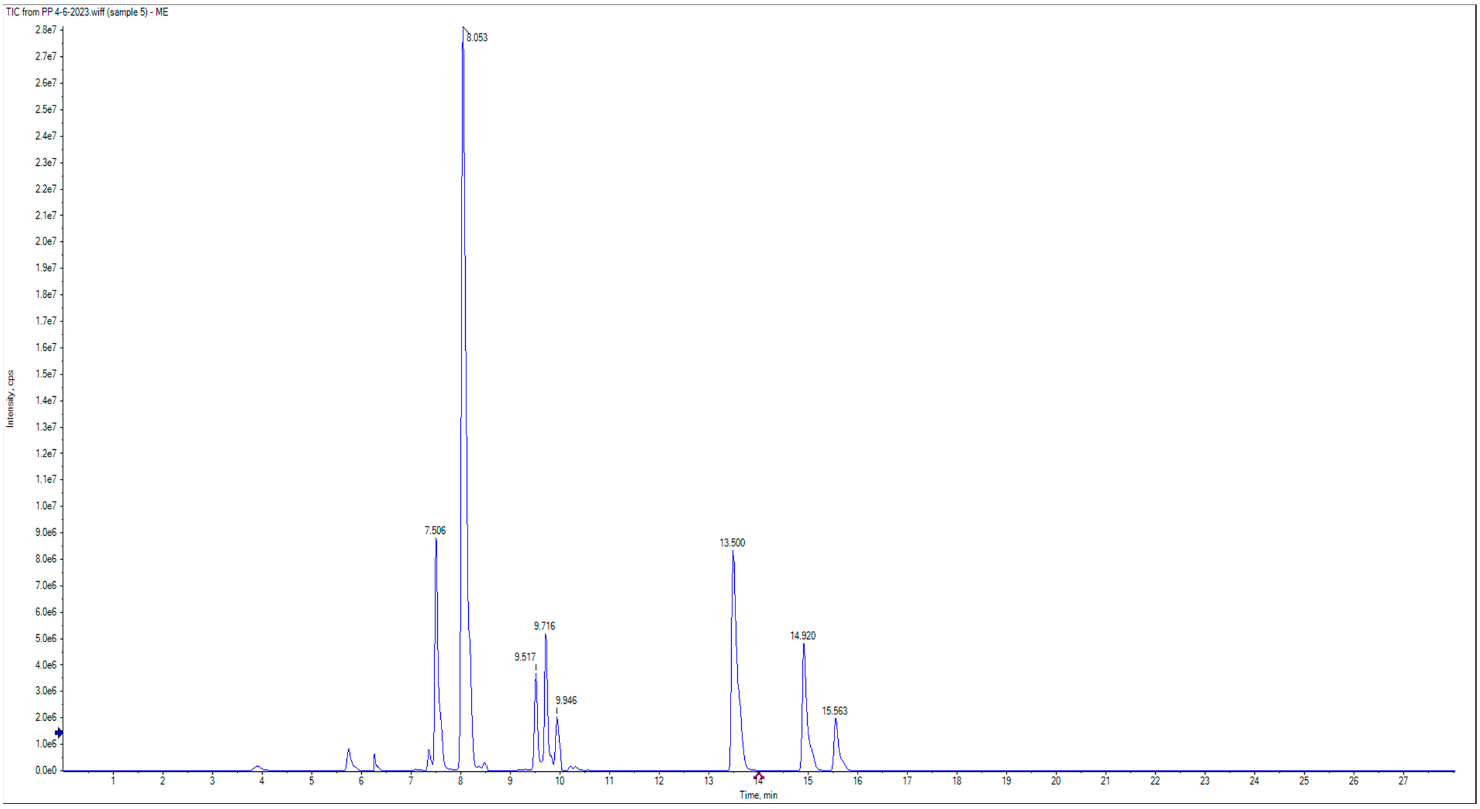

Figure S3. LC-ESI-MS spectra of mint extract.
